# Does the I-CARE e-learning programme improve primary care physicians’ competence in LGBTI health? Protocol for a two-arm, parallel-group, superiority randomised controlled trial in French-speaking Switzerland (EFFICARE study)

**DOI:** 10.1136/bmjopen-2026-119896

**Published:** 2026-08-04

**Authors:** Katja Bergonzoli, Gora da Rocha Rodrigues, María del Río Carral, Jelena Stanic, Xavier Mabire, Séverine Bochatay, Michael Amiguet, Yolanda Mueller, Patrick Bodenmann, Mauricio Avendano, Raphaël Bize

**Affiliations:** 1 Center for Primary Care and Public Health (Unisanté), University of Lausanne, Lausanne, Vaud, Switzerland; 2 HESAV School of Health Sciences—Vaud, HES-SO University of Applied Sciences and Arts Western Switzerland, Lausanne, Vaud, Switzerland; 3 Institute of Psychology, Faculty of Social and Political Sciences, University of Lausanne, Lausanne, Vaud, Switzerland; 4 Department of Social and Behavioral Sciences, Harvard T.H. Chan School of Public Health, Harvard University, Boston, Massachusetts, USA

**Keywords:** Health Equity, Vulnerable Populations, MEDICAL EDUCATION & TRAINING, Primary Care, Sexual and Gender Minorities, Randomized Controlled Trial

## Abstract

**Introduction:**

Lesbian, gay, bisexual, transgender or non-binary and intersex (LGBTI) populations experience persistent health inequities, including poorer mental and physical health outcomes and higher unmet healthcare needs compared with non-LGBTI populations. These inequities are partly attributable to insufficient training of primary care physicians (PCPs) in LGBTI health. However, evidence on effective and scalable training interventions for PCPs remains limited, particularly outside North America. This study evaluates the effectiveness of a self-directed e-learning programme—Improving Care and Access for Rainbow Equity (I-CARE)—designed to improve PCPs’ knowledge, attitudes, skills and behaviours related to LGBTI health.

**Methods and analysis:**

We will conduct a type I effectiveness-implementation hybrid, two-arm, parallel-group, superiority randomised controlled trial among PCPs practising in French-speaking Switzerland. Eligible physicians will be randomly assigned (1:1) to either the I-CARE programme (intervention) or an e-learning programme on motivational interviewing (control) matched for format and duration. Outcomes will be measured at baseline, immediately postintervention and at the 3-month follow-up. The primary outcomes are knowledge and attitudes towards LGBTI patients assessed at the postintervention time point using validated scales. Superiority will be declared if the intervention group is statistically significantly superior to the control group on at least one primary outcome and statistically significantly inferior on neither primary outcome. Secondary outcomes include skills, self-reported behaviours and the presence of LGBTI-inclusive environmental cues. Effectiveness will be analysed using generalised estimating equations. Implementation outcomes will be evaluated using the Reach, Effectiveness, Adoption, Implementation and Maintenance (RE-AIM) framework.

**Ethics and dissemination:**

Participants will provide electronic informed consent. All study data will be managed and stored on secure servers hosted by the Lausanne University Hospital (CHUV). All information will be handled in strict accordance with the Swiss Federal Act on Data Protection. The project was approved by the Central Ethics Commission of the University of Lausanne on 7 July 2025 (C_Services centraux_052025_00018). Study findings will be disseminated through peer-reviewed scientific publications and lay summaries intended for the public, Swiss PCPs and LGBTI communities.

**Trial registration number:**

ISRCTN93781961 (11 March 2026, updated 14 July 2026)

**STRENGTHS AND LIMITATIONS OF THIS STUDY**

- The randomised design with an active control programme matched for format, duration and continuing medical education credits allows any between-group difference to be attributed to content addressing the health of lesbian, gay, bisexual, transgender or non-binary and intersex (LGBTI) people rather than to attention or incentive effects.
- Stratified block randomisation protects against chance imbalance between arms on sex and age, two factors associated with physicians’ attitudes towards LGBTI patients.
- The use of validated, culturally adapted instruments and a 3-month follow-up enables reliable assessment of both immediate and sustained training effects.
- Reliance on self-reported outcomes may introduce social desirability bias, which partial blinding to the study hypothesis can only partly mitigate.
- Voluntary participation may limit the representativeness of the sample relative to all eligible primary care physicians.

## Introduction

 Lesbian, gay, bisexual, transgender or non-binary and intersex (LGBTI) individuals experience substantial health inequities compared with non-LGBTI individuals, including higher prevalence of mental health conditions, substance use, certain chronic diseases and barriers to healthcare access.^1–6^ Minority stress theory posits that structural, interpersonal and internalised stigma generate chronic stress, thereby contributing to adverse health outcomes.^7 8^ In Switzerland, 27% of LGBT people have experienced discrimination and violence in the healthcare setting because of their sexual orientation and/or gender identity,^6^ a pattern also observed in other countries.^9^ The proportion of LGBT people who forgo care due to a lack of trust in the healthcare system is more than two times that of the Swiss population (16.2% vs 6.9%).^6^ Implicit bias and lack of training among healthcare professionals (HCPs) have been hypothesised as important individual-level barriers contributing to the unmet health needs of LGBTI individuals.^10^

Although some HCP training programmes on LGBTQ+ health have been developed, evidence on their effectiveness is limited.^11^ A systematic review on educational interventions to reduce LGBTQ-related bias among medical, nursing and dental students and providers showed that bias-focused training programmes were associated with increased knowledge on LGBTQ healthcare issues.^12^ Another systematic review reported short-term improvements in knowledge and attitudes among healthcare students and professionals regarding LGBT-specific healthcare.^13^ A recent systematic review found that among 44 studies, improvement was shown among health professionals for knowledge (28 studies), attitudes (14 studies) and behaviours (seven studies).^11^ These studies, however, face important limitations. First, more than half of the studies had a sample size smaller than 100 and more than 80% of these studies were conducted in the USA, with only one being conducted in Europe. Second, most of the studies in these reviews did not involve a control group or used a randomised controlled experimental design, making it difficult to attribute changes to exposure to the programme. Third, many of these studies neither distinguished primary care physicians (PCPs) from other HCPs nor considered implementation outcomes (eg, adoption, acceptability).^14^

E-learning approaches offer scalable and cost-effective opportunities for continuing medical education (CME). A meta-analysis of studies on internet-based learning among health professions found that online training (on a wide range of medical topics) is as effective as traditional methods for improving knowledge, attitudes, skills and behaviours.^15^ A separate review of e-learning facilitators^16^ identified factors that predict effectiveness, including how user-friendly technologies were, flexibility of use, adaptation to the pace of learners, transferability of knowledge to clinical practice and the use of clinical case studies. On the other hand, lack of information technology (IT) skills was identified as a potential barrier to the use of e-learning methods by physicians.^17^

Using a randomised controlled trial design, we aim to evaluate the effectiveness of the Improving Care and Access for Rainbow Equity (I-CARE) programme in improving PCPs’ knowledge, attitudes, skills, self-reported behaviours and use of inclusive environmental cues related to LGBTI health. We hypothesise that, compared with an active control group receiving an e-learning programme on motivational interviewing (MI), I-CARE will lead to greater improvements in these outcomes. Additional implementation outcomes—reach, adoption, implementation fidelity and maintenance—will be assessed using the Reach, Effectiveness, Adoption, Implementation and Maintenance (RE-AIM) framework.

## Methods and analysis

### Study design and setting

This study is a type I effectiveness–implementation hybrid, two-arm, parallel-group, superiority randomised controlled trial with a 1:1 allocation ratio. It is conducted among PCPs practising in the French-speaking cantons of Switzerland (Fribourg, Geneva, Jura, Neuchâtel, Valais, Vaud and Bern), which comprise about one quarter of the Swiss population. Participants are individually randomised to receive either the I-CARE e-learning programme (intervention) or an active control e-learning programme on MI, matched for format and duration. Outcomes are assessed at baseline, immediately postintervention, and at the 3-month follow-up assessment. Participants are partially blinded to the specific study hypothesis but not to group allocation. The trial incorporates implementation outcomes evaluated using the RE-AIM framework.

### Participants

All PCPs holding eligible specialist titles and practising in the study region, as identified through a specialised private registry (estimated N=5601), will receive a postal invitation containing the study website address. Additional communication strategies may include announcements through cantonal medical societies, professional networks and conferences.

### Inclusion criteria

Eligible participants include PCPs practising at least 1-day per week in the study region, holding a recognised Swiss specialist title in general internal medicine, paediatrics, gynaecology, psychiatry or the ‘médecin praticien’ qualification (defined as physicians who have completed 3 years of postgraduate training but are not yet eligible for a specialist title).

### Exclusion criteria

Physicians who were involved in the development of either e-learning programme, who have previously been exposed to I-CARE materials or have already completed the MI e-learning programme offered to the control group, or who do not understand French, will be excluded.

### Study procedure

**Figure 1:**
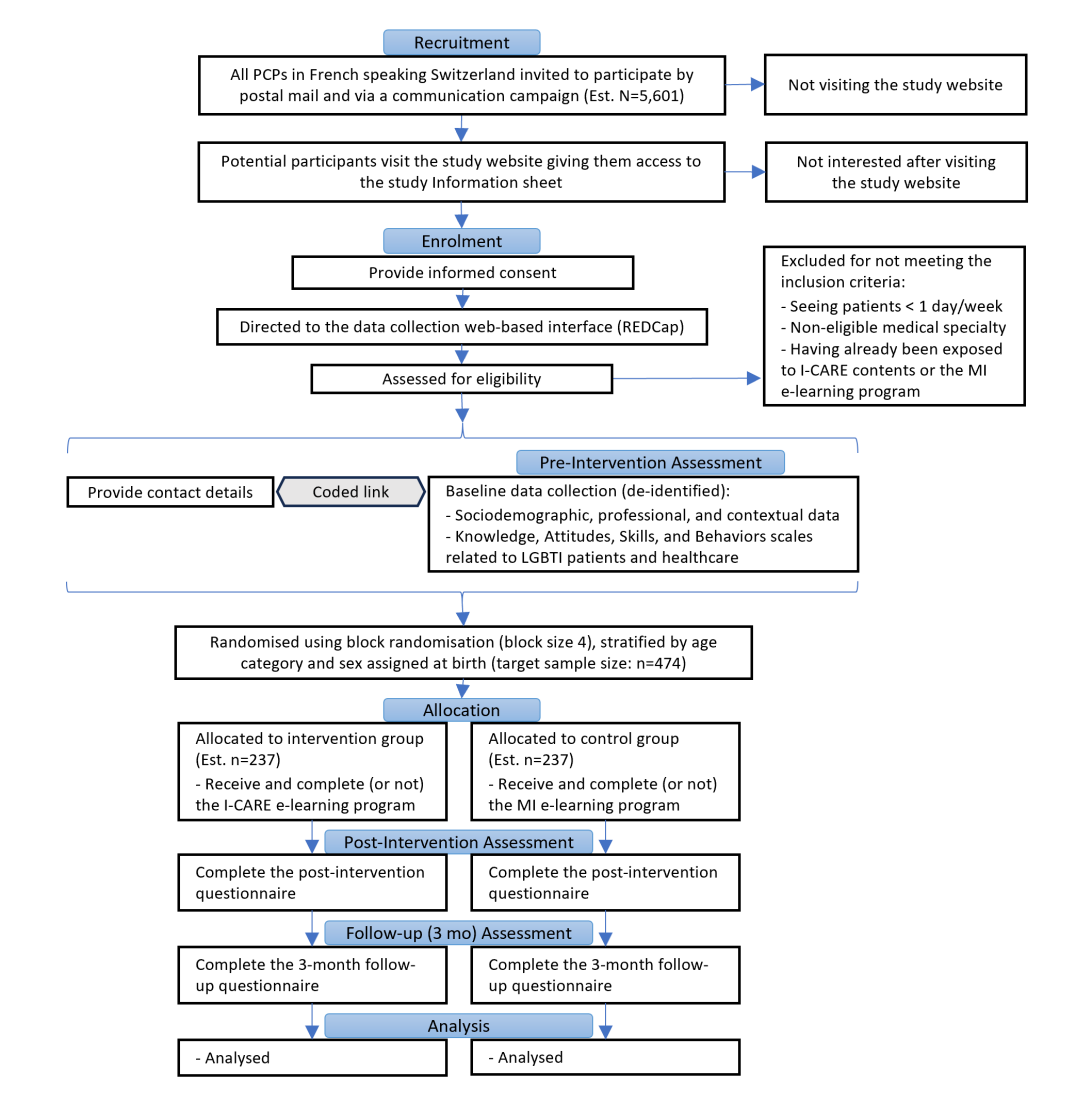
Study flow diagram. I-CARE, Improving Care and Access for Rainbow Equity; MI, motivational interviewing; PCPs, primary care physicians.

The study flow diagram is presented in figure 1. PCPs will access the study website to review the participant information sheet and provide electronic consent, after which they will complete a self-administered online baseline questionnaire. Participants will then be randomised to either the I-CARE (intervention) or MI (control) e-learning programme. The allocated programme will be accessible for 6 weeks, with regular automated reminders. Access to both e-learning programmes will be provided free of charge.

Once participants have completed their allocated e-learning programme—or, at the latest, 6 weeks after completing the baseline questionnaire if they do not complete the training programme—they will be invited to complete a self-administered online postintervention questionnaire. A final self-administered online follow-up questionnaire will be completed at the 3-month follow-up assessment. Participants who complete all questionnaires and the allocated e-learning programme will receive six CME credits as well as CHF 50 (about USD 64) in compensation. All questionnaires will be hosted online using REDCap (REDCap Consortium, Vanderbilt University, Nashville, Tennessee).^18 19^

All three questionnaires will assess effectiveness outcomes. The baseline questionnaire will also collect sociodemographic characteristics, while the postintervention questionnaire will assess participants’ satisfaction with the training programme. All appropriate measures have been taken to minimise participant errors during questionnaire completion, including prior cognitive testing and pilot testing, explicit drop-down response options, built-in skip logic, mandatory fields for key variables, standardised response formats and range and plausibility checks for numerical entries.

Recruitment began with the pilot phase on 10 July 2026 and is planned to continue until 31 December 2027. Data collection is expected to be completed in 2028, and the study is expected to be completed by 31 March 2029, including publication and dissemination activities.

### Pilot phase

To assess the feasibility of the study procedures and instruments under field conditions, we will conduct an internal pilot study.^20^ Five hundred PCPs randomly selected from the target population of French-speaking PCPs in Switzerland will be invited to participate in the pilot. The number of invited PCPs was chosen to allow estimation of key study process parameters with sufficient precision to inform progression to the main trial. In line with the internal pilot design, data collected during the pilot will be retained and included in the final trial dataset, provided that pilot findings do not lead to substantial modifications of the protocol, study procedures or outcome measurement instruments. The pilot will use the same design and study procedures as the main trial, except that recruitment will rely exclusively on postal invitations to sampled PCPs. Any changes to the protocol, study procedures or outcome measurement instruments will be communicated to the Central Ethics Commission of the University of Lausanne and described in subsequent publications.

#### Intervention and comparator

I-CARE is a novel self-directed e-learning programme designed to improve PCPs’ knowledge, attitudes, skills and behaviours related to LGBTI people and their healthcare needs. Its development was led by RB and PB in collaboration with PCPs, field experts, academic institutions (the Faculties of Medicine and University Hospitals of Lausanne and Geneva, and nursing schools), public health authorities in the canton of Vaud and French-speaking LGBTI community organisations.^21^ The programme comprises 23 video capsules (5–15 min each), organised into four thematic modules spanning three competency domains: knowledge—(1) introduction to sexual and gender diversity (terminology and key concepts, mechanisms of stereotyping, discrimination and violence, and specific life-course experiences) and (2) social determinants of LGBTI health (fundamental causes of health inequities, intersectionality, health status and access to care); attitudes—(3) inclusive communication in primary care (affirmative attitudes and language, and a welcoming and inclusive practice environment); and skills—(4) specific healthcare needs in primary care settings (sexual health history-taking; mental and relational health; and the specific needs of trans and non-binary people, intersex people, cisgender non-heterosexual people, older LGBTI people and rainbow families). A detailed description of the programme’s development and contents is available elsewhere.^21^ It is informed by the Minority Stress Model^22^ and the Cultural Competence in Healthcare Delivery framework,^23^ with a particular focus on cross-cultural communication and health equity.^11^ Completion of the programme requires approximately 3–5 hours.

Participants in the control group will follow a different e-learning programme that does not include specific content on LGBTI health but is comparable to I-CARE in format and duration. The control programme, *Entretien motivationnel: Cours de base pour médecins de premier recours* (University of Geneva), introduces MI for PCPs. This active control was preferred over a no-intervention condition to equalise exposure to e-learning content, engagement and incentives across arms, to minimise the risk of differential attrition and to support partial blinding to the study hypothesis. While MI promotes patient-centred communication skills applicable to all patients, including LGBTI patients, the programme contains no LGBTI-specific content, thereby preserving the contrast between arms on the outcomes of interest.

### Blinding

Participants will be informed that they are taking part in a study evaluating the effectiveness of two e-learning programmes for the CME of PCPs. In accordance with the approved ethics protocol, they will not initially be informed that the primary objective is to assess the effectiveness of the I-CARE programme specifically, in order to minimise response bias. PCPs will, therefore, be partially blinded to the study hypothesis, while remaining fully informed about the nature of the training they receive. Questions related to both training programmes, including MI and LGBTI healthcare, will be presented in a balanced manner.

### Randomisation

Each participant who completes the baseline questionnaire will be randomised (1:1) to either the intervention group, receiving an invitation and access to the I-CARE e-learning programme, or the control group. The randomisation will be stratified by sex assigned at birth (male or female) and age category, as physicians’ attitudes towards sexual and gender minorities have been shown to vary by gender and by generation, with earlier year of graduation and male gender predicting more stigmatising responses.^24 25^ Stratification protects against chance imbalance between arms on these prognostic factors. Three age categories will be used: PCPs born after 1980 (Generation Z and Millennials); PCPs born between 1965 and 1980 (Generation X) and PCPs born before 1965 (Baby Boomers generation or older ones if any). Allocation tables will be generated using Stata V.19 (StataCorp LLC, College Station, Texas) with block randomisation (block size of four) to ensure balanced distribution between the intervention and control groups.^26^ Participant allocation will be implemented automatically in REDCap.

### Primary and secondary outcomes

Two primary outcomes will be evaluated: knowledge and attitudes regarding LGBTI patients and their healthcare needs at the postintervention assessment. Superiority will be declared if the intervention group is statistically significantly superior to the control group on at least one primary outcome and statistically significantly inferior on neither primary outcome. Secondary outcomes include knowledge and attitudes at the 3-month follow-up assessment as well as skills in caring for LGBTI patients, self-reported behaviours towards LGBTI patients and the use of LGBTI-friendly environmental cues in clinical practice at both postintervention and 3-month follow-up assessments.

### Implementation outcomes

We will use the RE-AIM theoretical framework^27^ to evaluate implementation outcomes, as delineated in table 1.

**Table 1:** Implementation outcomes following the RE-AIM framework

	R Reach	E Effectiveness	A Adoption	I Implementation fidelity	M Maintenance
Type I effectiveness-implementation hybrid RCT	Proportion reached Representativity	Knowledge Attitudes Skills Behaviours	Proportion completing the I-CARE programme Satisfaction with the training programme	Technical incidents Time spent on the different learning activities Use of supplementary materials	Intention to continue to train on LGBTI health and healthcare Outcomes at the 3-month follow-up

I-CARE, Improving Care and Access for Rainbow Equity; LGBTI, lesbian, gay, bisexual, transgender or non-binary and intersex; RCT, randomised controlled trial.

The reach dimension will be assessed by comparing the number and sociodemographic characteristics of participants with those of the source population. Adoption will be evaluated based on the proportion of participants allocated to the I-CARE group who complete the training programme, along with postintervention items assessing participants’ satisfaction with the I-CARE content, format, ease of use and relevance to clinical practice.

### Data collection instruments

The primary outcomes, knowledge and attitudes, will be assessed using two subscales of the *QUeering Individual Relational and Knowledge Scales* for providers (QUIRKS-Provider).^28^ Reliability estimates based on McDonald’s omega support the unidimensionality of each subscale (knowledge: ω=0.75; attitudes: ω=0.69). Validity evidence indicates strong and statistically significant correlations between these subscales and the corresponding domains of *the Lesbian, Gay, Bisexual, and Transgender Development of Clinical Skills Scale* (LGBT-DOCSS), another validated instrument.^29^

Secondary outcomes will be assessed as follows. *Skills* will be measured using the *Clinical Preparedness* subscale of the LGBT-DOCSS. *Self-reported behaviours* will be assessed using 11 of the 15 items from the *Behaviour* subscale of the *Gay Affirmative Practice Scale* (GAP).^30^ Consistent with prior research,^31^ the wording of the GAP items was adapted to enhance inclusivity and contextual relevance (eg, replacing ‘gay/lesbian’ with ‘LGBTI’ and ‘clients’ with ‘patients’). Participants will also report on the presence of LGBTI-friendly environmental cues in their practice setting using the corresponding QUIRKS-Provider subscale (4 items).

The scales were translated and culturally adapted from English into French following the *International Society for Pharmacoeconomics and Outcomes Research* (ISPOR) guidelines,^32^ including two independent forward translations (one by a team member and one by a professional translator), followed by back-translation, comparison and harmonisation of the versions. Cognitive debriefing was conducted as a final step. All translations and modifications of the standardised scales and subscales were carried out in agreement with the original authors. The full translation report, including details of the modifications, is available on request.

### Sample size calculations

Based on previous research,^33^ we expect an effect size of Cohen’s d=0.31 on the Knowledge subscale of the QUIRKS-Provider scale. Using generalised estimating equations (GEE),^34^ and assuming 80% power, a two-sided significance level of 0.05, an intraindividual correlation of 0.4, and 20% loss to follow-up, we estimated that 474 participants would be required to detect this effect at the postintervention assessment. Recruiting this number of participants appears feasible, even with a low participation rate. As the marginal cost of including additional participants is negligible, no upper limit on the sample size has been set.

### Statistical analyses

We will first compare the two study groups with respect to sociodemographic characteristics and baseline measures of interest (knowledge, attitudes, skills, behaviours and environmental cues) to assess the extent of any baseline imbalance. We will then assess the representativeness of the study sample relative to the source population using data from Swiss medical registries.

Differences in the effectiveness outcomes over time and between study arms will be analysed using GEE.^35 36^ The model will include indicators for follow-up time points, study arm and their interaction, allowing estimation of differential changes in outcomes between groups at each time point. When appropriate, analyses will adjust for key baseline characteristics (eg, participating physicians’ sexual orientation and gender identity, which may be associated with baseline competence in LGBTI health) to improve precision and mitigate potential imbalance. The model is specified as follows:


(1) $$\mu_{it}=\beta_{0}+\beta_{T1}T1_{it}+\beta_{T2}T2_{it}+\beta_{IT1}(I_{i}\times T1_{it})+\beta_{IT2}(I_{i}\times T2_{it})+\gamma X_{i}$$

where

**Table:** 

$\mu_{it}$	is the expected value of the outcome measure for participant *i* at time *t*, where *t* corresponds to preintervention (*t* = 0), postintervention (*t* = 1) or the 3-month follow-up (*t* = 2);
$T1_{it}\;\text{and}\;T2_{it}$	are binary indicators for *t* = 1 and *t* = 2, respectively;
$I_{i}$	is a binary indicator of group assignment (0 = control, 1 = intervention);
$X_{i}$	represents adjustment covariates;
$\beta_{0}$	represents the baseline mean of the outcome measure under the model constraint of equal baseline means across groups;
$\beta_{T1}\;\text{and}\;\beta_{T2}$	represent the mean change in the control group from *t* = 0 to *t* = 1 and from *t* = 0 to *t* = 2, respectively;
$\beta_{IT1}\;\text{and}\;\beta_{IT2}$	represent the between-group differences in change from *t* = 0 to *t* = 1 and from *t* = 0 to *t* = 2, respectively;
γ	represents the regression coefficients associated with the covariates.

In this model, $\beta_{IT1}$ and $\beta_{IT2}$ estimate the intervention effect at the postintervention and 3-month follow-up assessments, respectively. In the presence of substantially different variability in the outcome at different time points, separate models for *t*=1 and *t*=2 may also be considered.

The model does not include separate baseline mean parameters for the two groups. This constraint is justified by randomisation, which implies equal expected baseline means across groups. Moreover, the corresponding unconstrained model has been shown to yield less efficient estimators of the intervention effect.^37^

GEE is a quasi-likelihood approach that provides consistent estimates of regression coefficients and valid inference in the presence of correlated observations, as occurs with repeated measurements within individuals over time. Unlike mixed models (MM), GEE allows direct specification of the working correlation structure and yields valid inference even if this structure is misspecified.^38^

A longitudinal data analysis (LDA) approach, such as GEE or MM, is preferable to an analysis of covariance (ANCOVA) framework, which treats the baseline value as a fixed covariate when modelling postintervention outcomes. One limitation of ANCOVA is that participants with missing baseline or postintervention outcome data are excluded from the analysis, which may reduce statistical power. By contrast, LDA methods use all available repeated measurements. ANCOVA may also underestimate the variance of adjusted group mean estimates because it does not fully account for variability in baseline values.^37 39^ However, there are warnings in the literature against the use of GEE with small sample sizes,^40^ whereas ANCOVA has been shown to have good performance even in small sample scenarios.^41^ ANCOVA might, therefore, be used in case the target sample size is not reached.

To assess the robustness of the findings, sensitivity analyses using MM or ordinary least squares with clustered standard error will be implemented.

Extent and patterns of missing data will be explored. Depending on the proportion of missing data, multiple imputation by chained equations may be implemented.

To address potential attrition, we will compare baseline characteristics of PCPs who complete all follow-up assessments with those of PCPs with missing follow-up data, separately within each study arm. In the presence of substantial differences, modified GEE approaches incorporating inverse probability weighting or multiple imputation will be considered.^42^

In addition to intention-to-treat estimates, we will estimate the complier average causal effect of the I-CARE programme using instrumental variable (IV) methods, where adherence is defined as completion of the full I-CARE programme. Randomised assignment to the I-CARE programme will be used as an IV for completion of the full I-CARE programme, with completion treated as the endogenous regressor and competency outcomes as the dependent variables. Under the standard IV assumptions, including that randomised assignment affects outcomes only through programme completion, IV estimates identify the causal effect of the I-CARE programme among compliers.

#### Additional research objectives

As a secondary research objective, we will assess heterogeneity in intervention effects by PCPs’ sex assigned at birth, gender identity, sexual orientation, age and specialty.

### Risk of bias

The effectiveness of the I-CARE programme will be assessed using self-reported outcomes, which may introduce social desirability bias. This bias would be of concern if it differed between study arms. Partial blinding and the use of validated data collection instruments are expected to mitigate this risk.

Another potential concern is non-random attrition. However, as the control programme (MI) will also be offered online free of charge and will provide a similar number of CME credits as the I-CARE programme, the risk of differential attrition is expected to be limited.

Although this does not compromise internal validity, external validity may be a concern if the final sample is not fully representative of the French-speaking PCP population in Switzerland. By analysing the reach dimension, we will describe the composition of the sample, assess differences from the source population and evaluate the extent to which the findings can be generalised.

Finally, the reliability of the attitudes subscale of the QUIRKS-Provider (ω=0.69) lies marginally below the conventional 0.70 threshold, which may attenuate the observed intervention effects on this primary outcome through non-differential measurement error.

### Data management and monitoring

During the project, data files will be managed and stored on secure servers hosted by Lausanne University Hospital (CHUV), with daily backups. All information will be kept strictly confidential in accordance with the Swiss Federal Act on Data Protection. At the end of the project, the file containing participants’ email addresses will be deleted. Deidentified data and metadata will be made available through the Unisanté data repository in accordance with the FAIR data principles and the Swiss National Science Foundation Open Research Data requirements. The Research Support Sector at Unisanté will provide quality control for data management and storage as well as for data curation and sharing.

### Ethics and dissemination

The study does not entail any specific risks or side effects for participants. Participation is voluntary, and participants may withdraw at any time without providing a reason and without any consequences. This right will be clearly stated in the information sheet prior to enrolment. All data will be coded. The correspondence between coded identifiers and email addresses will be known only to the Unisanté IT specialists responsible for the technical management of the REDCap interface, the principal investigator and the project’s postdoctoral researcher. Participants will also be informed that they may decline to answer sensitive questions. In the unlikely event of harm related to trial participation, appropriate measures will be taken in accordance with applicable Swiss legal and institutional requirements. The project was approved by the Central Ethics Commission of the University of Lausanne (CER-UNIL) on 7 July 2025.

A three-part communication strategy will be implemented to disseminate the objectives, findings and potential implementation of the I-CARE e-learning programme. The first component will target the public and will include a press conference, coverage in Swiss mainstream media and communication via social media. It will present the study findings and highlight the impact of the I-CARE programme on PCPs’ knowledge, attitudes, skills and behaviours in caring for LGBTI patients, with the aim of raising awareness of LGBTI health equity and reducing stigma.

The second component will target PCPs and the scientific community and will involve publication of scientific articles, presentations at medical conferences, podcasts and dissemination of newsletters through medical societies. We will also collaborate with the Swiss Health Network for Equity (https://www.health-equity-network.ch/en/), which has expressed its interest in disseminating the study findings through its network. Communication efforts will be tailored to motivate PCPs who have not yet participated in the I-CARE e-learning programme.

The third component will target LGBTI communities and will rely on cantonal and national LGBTI organisations to disseminate the study findings among LGBTI individuals in Switzerland. These communities will also be informed about the availability of a list of PCPs who have completed the I-CARE programme and consented to being publicly identified as such.

## Patient and public involvement

The perspectives of LGBTI people were incorporated into the research project through the involvement of LGBTI community representatives in a Scientific and Community Advisory Board. The board has been consulted during the development of the project and will continue to meet annually at key strategic stages.

In addition to representatives from LGBTI community and public health authorities, the board includes members from academic institutions with expertise in primary care, medical education, gender studies and gender diversity. This structure helps ensure that the intervention and research procedures are informed by community perspectives and remain relevant, acceptable and applicable to the populations and practice settings concerned.

## Importance of the study

Evidence indicates that LGBTI populations experience poorer health outcomes and multiple forms of stigma, which increase stress and contribute to elevated health risks. These risks are further compounded by barriers to accessing appropriate care, including discrimination, violence, implicit bias and insufficient training among HCPs. In Switzerland, where mandatory training in LGBTI health remains limited, a higher proportion of LGBTI individuals forgo medical care.

Although several studies have examined training programmes for HCPs designed to improve healthcare for LGBTI patients, most evidence originates from North America, with only one study conducted in Europe and none in Switzerland. Given that the effectiveness of such interventions is likely shaped by cultural, social and political contexts, context-specific evaluation is essential. Moreover, many existing studies are limited by small sample sizes and the absence of randomisation or control groups. Randomisation in the present study will allow causal inference regarding the effect of I-CARE on LGBTI care competencies. Partial blinding of the specific study hypothesis will further help reduce social desirability bias in self-reported outcomes.

By generating causal evidence on the effectiveness of e-learning programmes to improve PCPs’ competencies in LGBTI care, this study addresses an important research gap, particularly in the European context. It also contributes to broader educational and societal objectives by supporting improvements in professional training, with the goal of enhancing health outcomes and reducing health inequities among LGBTI populations.

Beyond the randomised controlled trial described in this article, the overall research project also includes qualitative work packages. As part of this broader research agenda, a sample of PCPs who complete the I-CARE programme within or outside the RCT, discontinue it, or are not interested in participating it in it will be interviewed to explore their experiences and perceived barriers. In addition, focus groups with nursing students, faculty members and practising nurses will examine how the I-CARE programme could be adapted to their professional contexts.
