# Supplementary material for: Does the I-CARE e-learning programme improve primary care physicians’ competence in LGBTI health? Protocol for a two-arm, parallel-group, superiority randomised controlled trial in French-speaking Switzerland (EFFICARE study)
Source: BMJ Open. 2026 Aug 4;16(8):e119896. doi: 10.1136/bmjopen-2026-119896 (PMC13448573; doi:10.1136/bmjopen-2026-119896)
Supplement: Supplementary file 1 [file bmjopen-2026-119896.reviewer_comments.pdf]

PEER REVIEW HISTORY

BMJ Open publishes all reviews undertaken for accepted manuscripts. Reviewers are asked to complete a checklist review form and are provided with free text boxes to elaborate on their assessment. These free text comments are reproduced below.

ARTICLE DETAILS

Title (Provisional)

Does the I-CARE e-learning program improve primary care physicians' competence in LGBTI health? Protocol for a two-arm, parallel-group, superiority randomized controlled trial in French-speaking Switzerland (EFFICARE study)

Authors

Bergonzoli, Katja; da Rocha Rodrigues, Gora; del Río Carral, María; Jelena, Stanic; Mabire, Xavier; Séverine, Bochatay; Amiguet, Michael; Mueller, Yolanda; Bodenmann, Patrick; Avendano, Mauricio; Bize, Raphaël

---

VERSION 1 - REVIEW

**Reviewer**	**1**
**Name**	**Whitfield, Darren**
**Affiliation**	**University of Maryland Baltimore, School of Social Work**
**Date**	**18-Jun-2026**
**COI**	**None**

Overall this protocol paper is well written and clearly outlines the study objectives. I have a few minor suggestions for strengthening the protocol.

The intervention could be describe a little better in terms of the content of the modules. It would help the reader understand the level of depth of the intervention content and assess the balance of the curriculum.

The control intervention; the MI module should be explained a little more in terms of how it relates to LGBTQ persons. Otherwise it is unclear how this is better than just not giving anything. Justify the control condition more.

I would move the description of the pilot on page 6 to before you describe the intervention and control condition since it occurs before the interventions. It would flow better.

The use of stratification in the randomization strategy is not well justified. It wasn't clear why this was important from the introduction. It might make sense to describe how age is related to treatment by primary care providers to set up this rationale.

The attitude subscale of the QUIRKS-Provider survey is weak based on the McDonald omega. I would allude to this as a potential limitation of the study. Lastly, will the study examine or control for sexual orientation of the primary care providers as research suggest LGBTQ primary care providers often have greater competence and their scores might impact the trial data. If so, it should be alluded to. If not, it should be listed as a potential limitation of the study.

VERSION 1 - AUTHOR RESPONSE

Reviewer 1	General comment: the protocol is well written and clearly outlines the study objectives; only minor suggestions are made to strengthen it.	We thank Dr Whitfield for his careful reading of our protocol and for his positive assessment. We respond to each of his suggestions point by point below.	None.	None
Reviewer 1	The intervention could be described in more detail regarding the content of the modules, to help the reader understand the depth of the content and assess the balance of the curriculum.	We agree that a brief overview of the module contents will help readers assess the depth and balance of the curriculum. We have added a sentence summarising the themes covered by each of the four thematic modules. A detailed description of the development and content of the I-CARE program is provided in the referenced development publication [21], which we now point to more explicitly.	**Before:** "The program comprises 23 video capsules (5–15 minutes each) organized into four thematic modules." **After:** "The program comprises 23 video capsules (5–15 minutes each), organized into four thematic modules spanning three competency domains: knowledge — (1) introduction to sexual and gender diversity (terminology and key concepts, mechanisms of stereotyping, discrimination and violence, and specific life-course experiences) and (2) social determinants of LGBTI health (fundamental causes of health inequities, intersectionality, health status, and access to care); attitudes — (3) inclusive communication in primary care (affirmative attitudes and language, and a welcoming and inclusive practice environment); and skills — (4) specific healthcare needs in primary care settings (sexual health history-	None

			taking; mental and relational health; and the specific needs of trans and non-binary people, intersex people, cisgender non-heterosexual people, older LGBTI people, and rainbow families). A detailed description of the program's development and contents is available elsewhere [21]."	
Reviewer 1	The control condition (MI module) should be explained further, including how it relates to LGBTQ persons; otherwise it is unclear how it is better than no intervention. The control condition should be better justified.	We thank the reviewer for the opportunity to clarify this methodological choice. An active control matched for format, duration and CME credits was preferred over a no-intervention control because it (1) equalises exposure to e-learning, engagement and incentives across arms, so that any between-group difference can be attributed to the LGBTI-specific content rather than to attention or participation effects; (2) minimises the risk of differential attrition in the control arm; and (3) supports partial blinding to the study hypothesis. The MI program was deliberately chosen because it is clinically relevant to consultations with all patients — including LGBTI patients — through its patient-centred communication approach, while containing no LGBTI-specific content, thereby preserving the contrast on the outcomes of interest. We have expanded the justification in the manuscript.	**Before:** "This program was chosen to ensure comparable exposure to educational content and to minimize the risk of differential attrition by offering clinically relevant content and the same number of CME credits."<br>**After:** "This active control was preferred over a no-intervention condition to equalise exposure to e-learning content, engagement and incentives across arms, to minimize the risk of differential attrition, and to support partial blinding to the study hypothesis. While motivational interviewing promotes patient-centred communication skills applicable to all patients, including LGBTI patients, the program contains no LGBTI-specific content, thereby preserving the contrast between arms on the outcomes of interest."	None
Reviewer 1	The description of the pilot (page 6) should be moved before the description of the intervention and control conditions, since the pilot occurs before the interventions; this would improve the flow.	We agree that this reordering improves the logical flow of the Methods section and have moved the 'Pilot phase' subsection accordingly.	We relocated the 'Pilot phase' subsection so that it precedes the 'Intervention and comparator' subsection. No changes were made to the	None

			wording of either subsection.	
Reviewer 1	The use of stratification in the randomisation strategy is not well justified; the introduction does not explain why this is important. Describing how age relates to treatment by primary care providers would set up this rationale.	We thank the reviewer for pointing out this gap. Stratification by sex assigned at birth and age category was chosen because physicians' attitudes toward, and familiarity with, LGBTI people and their healthcare needs have been shown to vary by gender and by age/generation. Stratified block randomisation protects against chance imbalance on these prognostic factors. We have added this rationale, with a supporting reference, to the 'Randomisation' subsection. Given the decision of minor revisions, we propose this targeted addition in the Methods rather than a restructuring of the Introduction.	**Before:** "The randomization will be stratified by sex assigned at birth (male or female), and age category."<br>**After:** "The randomization will be stratified by sex assigned at birth (male or female) and age category, as physicians' attitudes toward sexual and gender minorities have been shown to vary by gender and by generation, with earlier year of graduation and male gender predicting more stigmatizing responses [24, 25]. Stratification protects against chance imbalance between arms on these prognostic factors."	None
Reviewer 1	The attitudes subscale of the QUIRKS-Provider is weak based on McDonald's omega (ω = 0.69); this should be alluded to as a potential limitation of the study.	We agree with the reviewer. The reliability estimate of the attitudes subscale (ω = 0.69) lies marginally below the conventional 0.70 threshold. We selected the QUIRKS-Provider because it is one of the few instruments validated specifically for providers, with documented convergent validity against the LGBT-DOCSS. We now explicitly acknowledge this measurement limitation in the 'Risk of bias' section.	**We added this sentence** at the end of the 'Risk of bias' section: "Finally, the reliability of the attitudes subscale of the QUIRKS-Provider (ω = 0.69) lies marginally below the conventional 0.70 threshold, which may attenuate the observed intervention effects on this primary outcome through non-differential measurement error."	None
Reviewer 1	Will the study examine or control for the sexual orientation of the primary care physicians, given that LGBTQ providers often have greater competence and their scores might affect the trial data? If yes, this should be alluded to; if not, it should be listed as a limitation.	We thank the reviewer for raising this important point, which is indeed anticipated in our protocol. The baseline questionnaire collects participants' sociodemographic characteristics, including sexual orientation and gender identity, and pre-specified heterogeneity analyses by sexual orientation are described	In the second paragraph of the 'Statistical analyses' subsection:<br>**Before:** "When appropriate, analyses will adjust for key baseline characteristics to improve precision and mitigate potential imbalance."<br>**After:** "When appropriate, analyses will adjust for key	None

		under ‘Additional research objectives’ (“we will assess heterogeneity in intervention effects according to sex assigned at birth, gender identity, sexual orientation, age, and PCP specialization”). Randomization is expected to balance this characteristic across arms; in the event of baseline imbalance, the GEE models allow adjustment for it. We have made this more explicit in the second paragraph of the ‘Statistical analyses’ subsection and in the ‘Additional research objectives’ paragraph.	baseline characteristics (e.g., participating physicians’ sexual orientation and gender identity, which may be associated with baseline competence in LGBTI health) to improve precision and mitigate potential imbalance.”<br><br>In the ‘Additional research objectives’ paragraph:<br>**Before**: “As a secondary research objective, we will assess heterogeneity in intervention effects according to sex assigned at birth, gender identity, sexual orientation, age, and PCP specialization.”<br>**After**: “As a secondary research objective, we will assess heterogeneity in intervention effects by primary care physicians' sex assigned at birth, gender identity, sexual orientation, age, and specialty.”	
Manuscript’s authors	In our initial submission, we stated in the ‘ethics and dissemination’ subsection that the research team would not be able to directly link study data to individual participants. Following discussions between the research and IT teams, it became clear that two members of the research team — the principal investigator and the project's postdoctoral researcher — require access to this correspondence during data collection to provide participant support when needed and ensure the smooth conduct of the study. The Unisanté IT specialists responsible for the technical management of REDCap will also have access to this correspondence, as required to provide technical support. We revised the corresponding sentence in the manuscript accordingly. This change was approved by the UNIL Ethics Commission	None	In the first paragraph of the ‘ethics and dissemination’ subsection:<br>**Before:** “All data will be coded, and the research team will not be able to directly link study data to individual participants.”<br>**After:** “All data will be coded. The correspondence between coded identifiers and email addresses will be known only to the Unisanté IT specialists responsible for the technical management of the REDCap interface, the principal investigator, and the project’s postdoctoral researcher.”	None

	(C_Services centraux_052025_00018, 20 May 2026).			
Manuscript’s authors	We updated the estimated time required to complete the e-learning programs from 5–6 hours to 3–5 hours following additional testing.	None		We updated this information in the registry record
